# Assessing post-traumatic stress disorder in South African adolescents: using the child and adolescent trauma survey (CATS) as a screening tool

**DOI:** 10.1186/1744-859X-4-2

**Published:** 2005-01-31

**Authors:** S Suliman, D Kaminer, S Seedat, DJ Stein

**Affiliations:** 1 MRC Unit on Anxiety and Stress Disorders, Department of Psychiatry, University of Stellenbosch, Tygerberg, 7505, Cape Town, South Africa; 2 Department of Psychology, University of Cape Town, Private Bag Rondebosch, 7700, Cape Town, South Africa

**Keywords:** trauma, post-traumatic, stress, assessment, instruments

## Abstract

**Background:**

Several studies have demonstrated that South African children and adolescents are exposed to high levels of violent trauma with a significant proportion developing PTSD, however, limited resources make it difficult to accurately identify traumatized children.

**Methods:**

A clinical interview (K-SADS-PL, selected modules) and self-report scale (CATS) were compared to determine if these different methods of assessment elicit similar information with regards to trauma exposure and post-traumatic stress disorder (PTSD) in adolescents. Youth (n = 58) from 2 schools in Cape Town, South Africa participated.

**Results:**

91% of youth reported having been exposed to a traumatic event on self-report (CATS) and 38% reported symptoms severe enough to be classified as PTSD. On interview (K-SADS-PL), 86% reported exposure to a traumatic event and 19% were found to have PTSD. While there were significant differences in the rates of trauma exposure and PTSD on the K-SADS and CATS, a cut-off value of 15 on the CATS maximized both the number of true positives and true negatives with PTSD. The CATS also differentiated well between adolescents meeting DSM-IV PTSD symptom criteria from adolescents not meeting criteria.

**Conclusions:**

Our results indicate that trauma exposure and PTSD are prevalent in South African youth and if appropriate cut-offs are used, self-report scales may be useful screening tools for PTSD.

## Introduction

Adolescence is a critical developmental period that may also be characterized as a period of great risk to healthy development [1]. Adolescents are often subjected to a multitude of traumatic events in their daily lives. Those who are victimised and/or traumatised often lag behind those who are not, in terms of their behavioural and physical growth [2]. PTSD is one syndrome that may result from exposure to extreme trauma and is characterized by persistent reexperiencing, avoidance/numbing, and hyperarousal symptoms, present for more than one month and associated with significant distress and/or functional impairment [3].

Although community violence is highly prevalent in South Africa, a lack of awareness that children and adolescents may be adversely affected both in the short- and long- term [4], has contributed to a dearth of systematic data on youth exposed to violence and PTSD. Much of the work done has focused on the impact of political violence in the 1980's [5,6]. Although politically inspired violence has been in decline, criminal and domestic violence continues to prevail in local communities [7,8]. This has seen large numbers of children and adolescents being exposed to, and directly involved in, acts of violence [4]. For example, Peltzer's study on rural children in South Africa found that 67% had directly or vicariously experienced a traumatic event (elicited from direct interviewing of the child or from parent report) while 8% fulfilled criteria for PTSD [9].

Studies in the Western Cape have also noted high rates of traumatisation and PTSD among youth. In Cape Town, a retrospective chart review found PTSD to be one of the most common disorders at the Child and Adolescent Psychiatry Unit at Tygerberg Hospital [10]. In a community study in Khayelitsha, Ensink, Robertson, Zissis and Leger [11] used self-report measures to determine exposure to violence, as well as structured questionnaires and non-standardized clinical assessments to elicit symptoms and make psychiatric diagnoses in children aged 6 to 16 years. All children reported exposure to indirect violence. Ninety-five percent had witnessed violent events, 56% had experienced violence themselves, and 40% met the criteria for one or more DSM-III-R diagnoses. 22% met criteria for PTSD. The most commonly reported PTSD symptoms were: avoidance of thoughts and activities associated with the trauma, difficulties in sleeping, and hypervigilance.

A recent school survey of 307 Grade Ten pupils in the Western Cape, found that adolescents reported an average of 3.5 childhood traumatic experiences, and 12.1% met DSM-IV criteria for PTSD on self-report measures [12]. The most commonly reported symptoms were: avoiding thoughts about the event (34.4%), irritability (28.2%), difficulty showing emotion (26.5%), emotional upset at being reminded of the trauma (24.9%), and intrusive recollections of the event (19.4%). A significant positive correlation between multiple trauma exposure and PTSD symptoms was also found.

These aforementioned studies suggest that South African children, as a whole, are exposed to high levels of trauma and that a significant proportion develop PTSD. In order to develop preventative and ameliorative strategies for dealing with trauma, reliable and valid measurements of posttraumatic stress responses are needed. Although several instruments for assessing childhood disorders and symptoms have been developed over the past two decades [13], most have originated in the United States [14]. PTSD assessment instruments need preferably to be standardized in local samples to improve detection of the disorder. In South Africa, increasingly limited resources such as few school psychologists and large classrooms make it difficult to accurately identify traumatized children. Nevertheless, identification of children at risk for PTSD post-trauma may lead to the more efficient use of resources that are currently available.

The present study compared the psychometric properties of two instruments designed to assess trauma exposure and PTSD symptomatology and asked the question: " Do the K-SADS (a diagnostic clinical interview) and the CATS (a self-report scale) elicit similar information with regards to rates of trauma exposure and PTSD symptoms in a sample of South African adolescents?"

## Methodology

### Sample

A random sample of Grade 11 adolescents (n = 67) was selected from two Cape Town schools (36 from school A and 31 from school B). Of the 67 who were selected, 58 (17 males and 41 females) agreed to participate. Their mean age was 16 years, 8 months (SD: 0.59; range: 16–18 years). All spoke English as a first language. The majority of participants were non-White (n = 39 Coloured, n = 1 Asian, n = 18 White). The schools selected had participated in an aforementioned school survey of three schools that were conveniently sampled. Anonymous self-report questionnaires of trauma exposure and PTSD symptoms were utilized [12]. Lack of resources (time and money) did not allow for all participants in that study to be included in the present one.

### Instrumentation

#### 1. Demographic Questionnaire

This was clinician-administered and devised for the present study. It included information on age, sex, residential address, parental marital status, and occupation.

#### 2. Kiddie Schedule for Affective Disorders and Schizophrenia for School-Age Children – Present and Lifetime Version (K-SADS-PL) [15]

The K-SADS-PL is a standardised, DSM-IV based, clinician administered diagnostic interview, designed to provide an overview of current and lifetime psychopathology [16]. The K-SADS-PL has demonstrated good reliability and validity [17]. Abrosini [18] reported inter-rater reliability of 0.67 and 0.60 for current and lifetime PTSD, respectively. Construct validity [18] and criterion validity [19] have also been established.

Based on DSM-III-R and DSM-IV criteria, the K-SADS-PL has an initial 82 item screen interview that surveys key symptoms for current and past episodes of twenty different diagnostic areas, some of which screen for multiple disorders. Symptoms that have been present in the previous two months are recorded as current. For the purpose of this study, in order to make the K-SADS comparable to the CATS, PTSD symptoms judged to have been present in the past month were recorded as 'current'. Furthermore, only the PTSD and depression sections of the K-SADS-PL were administered.

The screen interview and diagnostic supplement format is unique to the K-SADS-PL and greatly facilitates administration of the instrument with normal controls and patient populations [16]. Most items on the K-SADS are rated on a zero to three point scale with a score of zero indicating no information is available; '1' suggesting the symptom is not present; '2' indicating sub-threshold levels of symptomatology; and '3' representing threshold criteria [15]. The Clinician-Administered PTSD Scale (CAPS-CA), arguably the current "gold standard" clinical interview for childhood PTSD, was not chosen for this study as it does not make use of screening questions for PTSD and is too lengthy to administer.

Although the K-SADS is designed to be administered to both parent and participant, it was administered to participants only. The reasons for this are twofold. First, our sample comprised older adolescents (16 to 18 years of age) and it was felt that the information gathered would be reasonably reliable. Second, as the primary objective of our study was to directly compare a clinician-assessment with a self-report, we did not consider it imperative that parental collateral be obtained. Previous studies have noted that parents may not always be aware of what their children are experiencing and may, therefore, not always be accurate historians [20].

#### 3. Child and Adolescent Trauma Survey (CATS) [21])

The CATS is a self-report index of PTSD qualifying stressors, non-PTSD life events, and PTSD symptoms. It is also a self-report measure of PTSD modelled on the Multidimensional Anxiety Scale for Children (MASC) [22] and the DSM-IV criteria for PTSD. The CATS is, however, not a DSM score scale but is derived using Item Response Theory (IRT). It includes stable indices of non-PTSD life events and provides a reliable and valid survey of secondary adversities, PTSD qualifying stressors, as well as a psychometrically sound symptom scale [21].

Unlike other self-rating scales, the CATS includes both a trauma exposure list and a PTSD inventory. Most self-rating scales focus on one or the other. The trauma list includes both direct (happened to me) and vicarious (happened to someone I know well) lifetime exposure. For example, the participant is required to indicate if s/he or someone s/he knows well has been badly beaten, or has been kidnapped during the participant's lifetime. Participants were also asked to note which was the worst event experienced and to report PTSD symptoms, experienced in the last month, in relation to this event.

In the PTSD section, participants are asked to rate how often in the past month they have had experiences that inventory the major symptom domains of PTSD – reexperiencing, avoidance and hyperarousal – on a four-point Likert scale. For example, participants are asked to indicate how often they are jumpy and nervous, or how often they sleep poorly – never, rarely, sometimes or often. Each of the DSM-IV PTSD criterion variables is represented by at least two questions [22].

According to March [23] a score of 27 and above on the PTSD scale should be taken to indicate that a child is at risk of clinically significant levels of PTSD. The CATS shows excellent internal [22] and test-retest reliability (March and Amaya-Jackson, unpublished data, 1997, [in [24]]).

Neither the K-SADS nor the CATS has been cross-culturally validated. However, the K-SADS is widely used internationally as a diagnostic measure in children and adolescents.

### Procedures

Permission to carry out the study was obtained from the Western Cape Department of Education and the Ethics Committees of the Universities of Stellenbosch and Cape Town. Consent from the heads of schools, parent bodies, and parents and learners was also obtained. Learners and their parents/guardians were informed that participation was entirely voluntary. Consent forms were handed to parents/guardians for signing prior to the interviews. Learners who opposed participation, or whose parents/guardians opposed participation, were excluded.

All evaluations were conducted in private in rooms allocated by school staff. The order of administration of the CATS and K-SADS was counterbalanced to control for practice effects. The evaluation per participant took approximately 45 minutes.

### Data Analysis

Microsoft Excel and Statistica software were used for data analysis. Student t-tests were used to determine significances for numeric data. The difference of two proportions test and the McNemar chi-square test were used to determine significances for categorical data. Cohen's kappa coefficients (K) were used to measure the level of agreement between the measures. As initial analysis revealed a statistically significant difference and low level of agreement between the measures, a more sensitive cut-off point was established for the CATS using a Receiver Operator Characteristic (ROC) Analysis.

## Results

### Exposure to Traumatic Events

On interview (Table 1), 86% of participants reported lifetime exposure to at least 1 traumatic event, (mean = 2.3; SD = 1.7; range = 0–10), while on self-report (Table 2), 91% of participants reported direct or indirect lifetime exposure to at least 1 traumatic event (mean = 3.7; SD = 3.2; range = 0–14). The difference of two proportions test revealed that the number of participants who reported experience of a traumatic event on each measure was not significantly different (p = 0.36). The level of agreement between the measures was 0.74 (SE = 0.15; CI = 0.46–1.0). These events were random, rather than politically-motivated experiences of trauma.

**Table 1:** Frequencies of reported traumas on the K-SADS

**Event**	**Number**	**%**
Car accident	4	6.9
Other accident	9	15.5
Fire	2	3.4
Witness of a disaster	4	6.9
Witness of a violent crime	14	24.1
Victim of a violent crime	6	10.3
Confronted with traumatic news	33	56.9
Witness to domestic violence	18	31
Physical abuse	2	3.4
Sexual abuse	5	8.6
Other	11	19

(n = 58)

**Table 2:** Frequencies of reported traumas on the CATS

**Event**	**Happened to Me**		**Happened to Someone I Know Well**	
	
	**Number**	**%**	**Number**	**%**
Badly bitten by a dog or another animal	8	13.8	15	25.9
Badly scared or hurt by a gang or criminal	4	6.9	17	29.3
Badly beaten	1	1.7	14	24.1
Shot or stabbed	0	0	16	27.6
Terrible fire or explosion	0	0	7	12.1
Chemical or other deadly poisoning	1	1.7	4	6.9
Bad storm, flood, tornado, hurricane or earthquake	2	3.4	6	10.3
Bad car, boat, bike, train, or plane accident	3	5.2	18	31
Other very bad accident	5	8.6	9	15.5
Got sick and almost died or died	5	8.6	28	48.3
Kidnapped or held captive	0	0	5	8.6
Suicide attempt or died from suicide	4	6.9	19	32.8
			
I was taken away from my family	1	1.7		
I saw something terrible happen to a stranger	16	27.6		
			
Other shocking or terrifying event	5	8.6	2	3.4

(n = 58)

### Differences in Reporting of Trauma Exposure Between Measures

When both direct ("happened to me") and vicarious ("happened to someone I know well") trauma exposure on the CATS was considered, significantly more traumas were endorsed on the CATS (mean = 3.7) than on the K-SADS (mean = 2.3) (t = -3.94; p = < 0.01). However, when vicarious exposure was excluded on the CATS, the number of traumas reported on the K-SADS was significantly higher (t = 5.68; p = < 0.01).

### PTSD Diagnoses

11 participants (19%) received a diagnosis of PTSD on the K-SADS, while only 1 participant (1.7%) received a diagnosis of PTSD on the CATS using a cut-off of 27, as suggested by March [23]. This difference was significant (χ^2 ^= 50.3; p < 0.01) with the level of agreement between the measures (K) being 0.14 (SE = 0.25; CI = -0.35–0.62). Three participants diagnosed with PTSD(27.3%) on the K-SADS appeared to have developed it in response to sexual assault trauma, as did the one participant screened with PTSD on the CATS.

### ROC Analysis

Given the low level of agreement using a CATS cut-off of 27, an ROC analysis (Table 3) was done in order to establish a CATS cut-off score that would be more appropriate for the sample. First, using the K-SADS as the "gold" standard for a diagnosis of PTSD (a measure that identifies those individuals who have or do not have a disorder), the sensitivity and specificity for various CATS cut-off scores were established. In addition to *sensitivity *(the proportion of true positives that are test positives [true positive probability]) and *specificity *(the proportion of true negatives that are test negatives [true negative probability]); '*1 – specificity*' (false positive probability), the *gradients *between each point, and the *positive and negative predictor values *were calculated (the predictive value of a positive test is the proportion of those with a positive test result who actually have the disorder, while the predictive value of a negative test is the proportion of those with a negative test result who do not have the disorder).

**Table 3:** Receiver Operator Characteristic (ROC) and Predictive Values

**Cut-off Values**	**Sensitivity**	**Specificity**	**1-specificity**	**Gradient**	**Predictive Values**	
					**Positive**	**Negative**
0	100	0	100	--	--	--
1	100	2	98	0	22	100
3	100	17	83	0	22	100
4	91	21	79	2.25	21	91
5	91	23	77	0	22	92
7	91	28	72	0	23	93
8	91	32	68	0	24	94
9	91	36	64	0	25	94
10	82	36	64	∞	23	90
11	82	40	60	0	24	91
12	82	47	53	0	27	92
13	82	53	47	0	29	93
14	82	62	38	0	33	94
15	73	70	30	1.12	36	92
16	64	81	19	0.82	44	91
17	64	87	13	0	44	91
18	55	87	13	∞	50	89
19	55	92	8	0	60	90
20	55	94	6	0	67	90
21	55	98	2	0	86	90
22	55	100	0	0	100	90
23	36	100	0	∞	100	87
25	18	100	0	∞	100	84
27	9	100	0	∞	100	83

**Figure 1:**
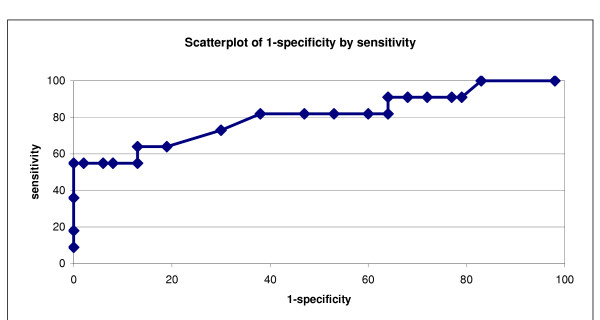
ROC curve

An ROC curve graph (sensitivity and 1 – specificity) was also plotted (Figure 1). The area under the curve (sensitivity of the scale) was found to be 0.805. A cut-off that gives a gradient closest to 1 is usually chosen as appropriate because it maximises both sensitivity and specificity. With a cut-off of 15, 22 participants had scores indicative of PTSD on the CATS. However, the difference between the number of participants diagnosed with PTSD on the K-SADS and the CATS remained significant (χ^2 ^= 19.9; p < 0.01). While significance was in the expected direction (i.e. the prevalence on self-report was higher than on interview), but the level of agreement was doubled (K = 0.31; SE = 0.14; CI = 0–0.59).

A t-test comparing the scalar scores of participants with a PTSD diagnosis on the K-SADS (mean = 18.5, SD = 7.8) and participants without a PTSD diagnosis (mean = 10.4, SD = 6.4) showed the difference between the two measures to be significant (t = 3.64; p < 0.01).

### PTSD Symptom Clusters

On the K-SADS, 18 participants met DSM-IV criteria for re-experiencing symptoms (Criterion B), 15 participants met criteria for avoidance symptoms (Criterion C), and 18 participants met criteria for hyperarousal symptoms (Criterion D). Since the CATS is not a DSM-IV PTSD score scale, the number of participants meeting individual DSM-IV criteria could not be established. However, the CATS does include six items for Criterion B, two for Criterion C, and four for Criterion D, so a scalar score for each of these factors could be derived. Student t-tests comparing mean Criterion B, C, and D CATS scalar scores for participants fulfilling criteria B, C, and D, respectively, on the K-SADS, with those not meeting criteria, revealed significant differences for all three symptom clusters at the 0.05 level. The Criterion B mean scalar score for participants meeting Criterion B on the K-SADS was 7.3 (SD = 4.3) compared to 4.8 (SD = 3.1) for those not meeting criteria (t = -2.45; p = 0.02). Participants with Criterion C on the K-SADS had a mean scalar score of 3.9 (SD = 1.8), while those not meeting Criterion C had a mean score of 2.2 (SD = 2.0) (t = -3.12; p = 0.03). The mean scalar score for participants meeting Criterion D was 5.1 (SD = 3.3) compared to a mean score of 3.1 for those not meeting this criterion (SD = 2.5) (t = -2.50; p = 0.02).

### PTSD Symptoms

Table 4 compares the percent endorsement of PTSD symptoms on the K-SADS and the CATS. Student t-tests were used to compare number of symptoms reported on the K-SADS (mean = 3.3, SD = 5.0) with number of symptoms reported on the CATS (mean = 3.7, SD = 2.8). No significant differences were noted (t = -0.83; p = 0.41). Kappa's were then done to measure the level of agreement between the measures for symptoms that could be directly compared for the sample as a whole, and for participants with and without PTSD on the K-SADS (Table 5). Items assessing sleep problems, distress at reminders of event, and exaggerated startle responses evidenced the best agreement across instruments.

**Table 4:** PTSD symptoms

**Rate of PTSD symptoms on the K-SADS**		**Rate of PTSD symptoms on the CATS**	
symptom	%	symptom	%

Comparable Symptoms			

Recurrent thoughts or images of events	28	I go over and over what happened in my mind	40
Efforts to avoid thoughts or images associated with the trauma	28	I try not to think about what happened	47
Insomnia	22	I sleep poorly	26
Irritability or outbursts of anger	24	I am grouch and irritable	36
Distress at reminders of event	16	When something reminds me of what happened I get tense and upset	21
Exaggerated startle response	17	I am jumpy and nervous	29
Nightmares	16	I have bad dreams about what happened	9
Difficulty concentrating	19	I have trouble keeping my mind on things	28
Efforts to avoid activities or situations that arouse recollections of the trauma	21	I try to stay away from things that remind me of what happened	21

Non-comparable Symptoms			

Sense of foreshortened future	3	I worry that what happened will happen again	57
Feelings of detachment or estrangement	21	I get scared when I think about what happened	38
Inability to recall important aspects of the trauma	10	I have unwanted thoughts about what happened	21
Restricted affect	28		
Hypervigilance	17		
Physiological reactivity upon exposure to reminders	9		
Dissociative episodes, illusions or hallucinations	21		
Diminished interest in activities	22		
Repetitive play related to event / reenactment	2		

**Table 5:** Levels of agreement for comparable PTSD symptoms

**PTSD Symptom**				**95% Confidence Interval**	
				
		**Observed Kappa**	**Standard Error**	**Lower Limit**	**Upper Limit**
**Recurrent thoughts or images of event**	(i)	0.02	0.14	-0.31	0.26
	(ii)	-0.57	0.22	-1.01	-0.13
	(iii)	0.01	0.18	-0.33	0.35
**Trying not to think about the event**	(i)	0.25	0.13	-0.003	0.51
	(ii)	-0.14	0.56	-1.24	0.96
	(iii)	0.07	0.17	-0.26	0.41
**Sleep problems**	(i)	0.44	0.15	0.15	0.72
	(ii)	0.61	0.25	0.11	1
	(iii)	0.16	0.23	-0.3	0.62
**Anger and irritability**	(i)	0.24	0.14	-0.05	0.52
	(ii)	0.24	0.3	-0.35	0.83
	(iii)	0.13	0.19	-0.24	0.49
**Distress at reminders of event**	(i)	0.48	0.16	0.17	0.79
	(ii)	0.44	0.28	-0.1	0.98
	(iii)	0.17	0.29	-0.4	0.74
**Exaggerated startle response**	(i)	0.39	0.15	0.09	0.68
	(ii)	0.3	0.35	-0.38	0.98
	(iii)	0	0.3	-0.59	0.59
**Nightmares**	(i)	0.2	0.23	-0.26	0.65
	(ii)	0.23	0.26	-0.28	0.73
	(iii)	-0.05	0.45	-0.93	0.82
**Difficulty concentrating**	(i)	0.19	0.17	-0.04	0.51
	(ii)	0.35	0.26	-0.15	0.86
	(iii)	0.03	0.23	-0.41	0.48
**Efforts to avoid reminders of event**	(i)	0.27	0.15	-0.03	0.56
	(ii)	-0.31	0.3	-0.89	0.28
	(iii)	0.34	0.18	-0.02	0.7

(i) Total sample (N = 58); (ii) Participants with PTSD on the K-SADS (N = 11);

(iii) Participants without PTSD on the K-SADS (N = 47).

Participants with and without a diagnosis of PTSD based on the K-SADS were compared on percentage endorsement of each CATS symptom. The difference of two proportions test showed a significant difference in only five of the twelve symptoms (recurrent thoughts about the event, exaggerated startle response, difficulty concentrating, avoidance of physical reminders of the event, and nightmares). The other symptoms did not discriminate well between participants with and without PTSD.

### Internal Consistency

Alphas of 0.96, 0.97 and 0.93 were obtained for the K-SADS PTSD Criterion B, C, and D respectively. These were not improved by the removal of any items within a symptom category (Criterion B, C, and D).

Alphas of 0.79 and 0.67 were obtained for Criteria B and D in the CATS, which were not improved by the removal of any items. An alpha was not calculated for Criterion C as there are only two items comprising that category.

A total internal consistency of 0.99 was obtained for the PTSD section of the K-SADS and a total internal consistency of 0.86 was obtained for the CATS.

## Discussion

Compared with other international community-based studies [e.g. [25,26]], our study found high rates of trauma exposure on both clinician-administered and self-report measures in adolescents, with the majority (86% on the KSADS and 91% on the CATS) reporting exposure to at least one traumatic event in their lifetime. These rates are consistent with previous South African studies [e.g. [12]].

Consistency in reporting of traumatic events was low between the measures and participants were more likely to endorse a trauma on the CATS than on the K-SADS. This may be attributable to the fact that more vicarious traumatisation as compared to directly experienced or witnessed traumas is asked about in the CATS, or to the relative privacy of the self-report format- participants may have felt more comfortable in admitting to traumatic experiences on a self-report scale which may be perceived as less intrusive [27].

19% of adolescents in the sample were diagnosed with PTSD on the K-SADS. This rate is comparable with the PTSD rate found in a larger sample of adolescents who were sampled in the same geographical region [28]. The rate of 19% is, however, higher than that documented in a previous survey of which this sample constituted a sub-sample [12] The passage of time (i.e. more than a year between assessments) may be one reason for the higher rate of PTSD in the sub-sample. Most other South African community-based studies in adolescents (with the exception of a study by Ensink et al. [11], that have used self-report measures of assessment, have documented lower rates of PTSD than was found in this study.

The differing rates of PTSD between the K-SADS and the CATS (using a cut-off 27 on the CATS), suggests that this cut-off may be too high in our setting. The ROC analysis yielded a cut-off of 15 on the CATS. This cut-off maximizes both the number of true positives and true negatives and may be more appropriate. Using a cut-off of 15, 22 participants (38%) were diagnosed with PTSD. While there still remained significant differences in the rates of PTSD using this cut-off, the level of diagnostic agreement was higher than with a cut-off of 27. Our findings are consistent with studies that have demonstrated that self-report measures [e.g. [29,30]] yield higher rates of psychiatric diagnoses than clinician-based interviews [e.g. [25,27]]. Moreover, significant differences in CATS severity scores between participants with and without PTSD, suggests that the CATS discriminates well between those with and without the disorder.

Further, significant differences were found between mean CATS scores for Criterion B (intrusive), C (avoidance), and D (hyperarousal) symptoms between participants meeting DSM-IV criteria for these clusters on the K-SADS, and those not meeting criteria. The two symptoms that were most frequently endorsed on both the K-SADS and the CATS (recurrent thoughts/ images of event and efforts to avoid thoughts of the event) are also among the symptoms most frequently reported in other studies [11,12], suggesting that careful inquiry of these symptoms is important. However, the level of agreement for specific symptoms appeared to be suboptimal: overall, participants who reported symptoms on the K-SADS did not necessarily report the same symptoms on the CATS. That said, participants with PTSD were more consistent in their reporting than those without PTSD. Nevertheless, the lack of significant differences in the numbers of symptoms reported between the measures suggests that these measures may be comparable in eliciting the average number of symptoms experienced post-trauma. The CATS appeared to discriminate well between those with and without PTSD on five of twelve items (recurrent thoughts about the event, exaggerated startle response, difficulty concentrating, avoidance of physical reminders of the event, and nightmares), suggesting that these symptoms may be more sensitive indicators of PTSD.

### General Implications of Findings

The K-SADS and CATS yield different information about the level and type of trauma exposure, therefore researchers and clinicians should be cautious when substituting one for the other. The K-SADS is likely to yield more detailed information on witnessing traumatic events, while the CATS is likely to yield more information on vicarious trauma exposure. Adolescents are also more likely to endorse a trauma on the CATS than they are on the K-SADS. The significantly larger proportion of adolescents with scores indicative of PTSD on the CATS, compared to the K-SADS, indicates that the CATS may be better utilized as a PTSD screening device (as suggested by its author), with a cut-off threshold of 15 instead of the original threshold of 27, in the South African context. This will identify over one third of all participants with PTSD while making few false positive identifications. This will, however, require replication in a larger South African cohort. For an actual PTSD diagnosis, a clinician-based diagnostic interview may be more appropriate even though it is likely to be more time consuming.

Several limitations are worth mentioning. First, the K-SADS was not administered to both parents and learners as it is intended to be, thus participants' responses were not verified by collateral information from parents and legal guardians. Second, the sample comprised predominantly female adolescents of mixed race. Even though this constitutes the majority ethnic group in the province, the small sample and truncated age limits the generalizability to the larger population. Further, socio-demographic variables (e.g. social class, family income and race) were not accounted for in the analysis. Third, cultural influences may favour certain symptoms of trauma over others [31] and it has been noted that there is a need to identify other post-traumatic expressions of distress, such as somatization [32,33]. Both the K-SADS and the CATS do not attempt to capture these experiences. However, PTSD has been widely documented in traumatized cohorts from different ethnocultural backgrounds and those from non-Western cultures who meet PTSD diagnostic criteria often show a similar clinical course and response to treatment [33]. Fourth, we used the DSM-IV concept of trauma to compare these instruments and some authors, for example Summerfield [34], have highlighted some of the difficulties with the concept of trauma as defined in the DSM. It may be that events counted and endorsed as traumas were too broad to ascertain their level of agreement on the K-SADS and the CATS. Fifth, while we attempted to compare traumatic events and symptoms across instruments, it must be noted that these instruments are not necessarily suited to direct comparison. For example, the two instruments measure different traumatic events, automatically placing a cap on the level of agreement.

In view of the high levels of violence in South African youth, identification of those children and adolescents with PTSD is important and necessary to allow for appropriate interventions. Owing to limited resources, administration of diagnostic clinical interviews to all youth is not feasible. Self-report scales, even though they do not replace clinical interviews, may be useful in identifying those youth in the community who are most at risk. This may help to facilitate more targeted and efficient treatments. While this study has limitations, some tentative conclusions can nevertheless be drawn. High rates of trauma exposure and PTSD characterize South African children and adolescents. Self-report scales may be better utilized as screening instruments rather than as diagnostic tools. To establish more efficient ways of diagnosing PTSD and other post-traumatic sequelae in the South African setting, future studies (using self-rating scales and brief PTSD diagnostic measures) should be conducted in larger samples, more representative of the South African population. In particular, we need to establish and verify more suitable cut-off values on these instruments to enable the identification of those children and adolescents who are at higher risk for PTSD and other disorders.

## Competing Interests

The author(s) declare that they have no competing interests.
